# Splenic Rupture Diagnosed with Bedside Ultrasound in a Patient with Shock in the Emergency Department Following Colonoscopy

**DOI:** 10.5811/westjem.2015.6.27548

**Published:** 2015-10-20

**Authors:** William Mulkerin, Tsuyoshi Mitarai, Laleh Gharahbaghian, Phillips Perera

**Affiliations:** * Stanford University Medical Center, Department of Emergency Medicine, Fellow Physician, Stanford, California; † Stanford University Medical Center, Department of Emergency Medicine, Clinical Assistant Professor, Stanford, California; ‡ Stanford University Medical Center, Department of Emergency Medicine, Clinical Associate Professor, Stanford, California

A 64-year-old male presented to the emergency department (ED) with near syncope and worsening left flank and shoulder pain. He had undergone a difficult colonoscopy two days prior due to a tortuous colon. Initial vital signs were normal. He looked uncomfortable and had significant left upper quadrant abdominal tenderness with guarding. Thirty minutes after ED arrival, his blood pressure dropped to 73/59 mmHg, requiring aggressive fluid resuscitation. Bedside focused assessment with sonography in trauma (FAST) exam demonstrated free fluid in the abdomen with mixed echogenicity of the spleen, suggestive of splenic injury. Computed tomography (CT) demonstrated a large subcapsular splenic hematoma with active extravasation and surrounding intraperitoneal free fluid (Figure, Video). He was admitted to the surgical intensive care unit. Hemorrhage continued after interventional radiology performed embolization of the splenic artery. He then required laparoscopic splenectomy on hospital day 2 to control bleeding. He subsequently did well and was discharged on hospital day 10.

Colonoscopy has been associated with spontaneous intra-abdominal organ injury. Jammal et al described a case of subcapsular liver hematoma associated with colonoscopy.^1^ Lauretta et al described a case of splenic rupture following colonoscopy that had a delayed diagnosis due to initial physician consideration of alternate pathology, specifically intestinal rupture.^2^ Shankar and Rowe described a case of splenic injury following colonoscopy and then reviewed the literature, finding a total of 93 cases.^3^ Increasing age was found to be a risk factor. The authors also emphasized that patients may present with only moderate abdominal pain. A larger case series in 2012 by Aubrey-Bassler and Sowers described 613 case of splenic rupture without known risk factors. Of these, 327 occurred as a presenting manifestation of an underlying disease. Infections were found in 143 patients, with malaria most common (65 cases) and mononucleosis second (42 cases). Medical procedures were also highly associated with splenic rupture, and 87 cases were associated with colonoscopy.^4^ In 2011 Fishback et al described the clinical and CT findings of 11 patients with splenic rupture after colonoscopy. CT demonstrated splenic injury with subcapsular hematoma and/or perisplenic hematoma in 10 cases and hemoperitoneum in eight cases.^5^ Singla et al found 102 patients with splenic injury following colonoscopy.^6^ The majority (73 patients) required operative intervention with 96% requiring splenectomy. This information should prompt emergency physicians to consider splenic injury in patients presenting with abdominal pain (even mild) and/or shock in those who have recently undergone a colonoscopy. Bedside point-of-care ultrasound using the FAST exam may be effective in initially identifying these patients, especially those in shock requiring immediate resuscitation and surgical consultation like the patient discussed in this case.

## Figures and Tables

**Figure:**
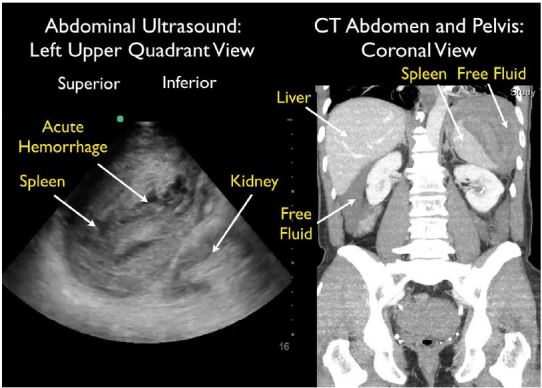
Splenic rupture after colonoscopy. Left image, ultrasound. Right image, computed tomography scan (CT).

**Video:** Spleen rupture case.
